# Performance Analysis of Bloom Filter for Big Data Analytics

**DOI:** 10.1155/2022/2414605

**Published:** 2022-12-22

**Authors:** Suliman A. Alsuhibany, Mohammed Alsuhaibani, Rehan Ullah Khan, Ali Mustafa Qamar

**Affiliations:** ^1^Department of Computer Science, College of Computer, Qassim University, Buraydah, Saudi Arabia; ^2^Department of Information Technology, College of Computer, Qassim University, Buraydah, Saudi Arabia

## Abstract

The rapid rise of data value, such as social media and mobile applications, results in large volumes of data, which is what the term “big data” refers to. The increased rate of data growth makes handling big data very challenging. Despite a Bloom filter (BF) technique having previously been proposed as a space-and-time efficient probabilistic method, this proposal has not yet been evaluated in terms of big data. This study, thus, evaluates the BF technique by conducting an experimental study with a large amount of data. The results revealed that BF overcomes the efficiency not present in the space-and-time of indexing and examining big data. Moreover, to address the increase of false-positive rate in using BF with big data, a novel false-positive rate reduction approach is proposed in this paper. The initial experimental results of evaluating this method are very promising. The novel approach helped to reduce the false-positive rate by more than 70%.

## 1. Introduction

The fast growth of data value, such as social media and mobile applications, leads to generating huge volumes of data. Thus, methods for gathering structured and unstructured digital information, which form big data, have been developed [1]. Big data has several applications, such as in social networks and healthcare. A recent survey by the authors of reference [2] indicates that proficiently handling big data is competitive and challenging. This challenge centers mainly on big data security analytics (BDSA) because of a rapid increase in the type of cyber-attacks [3]. While deeply testing the networks' packet is central [4], correlating events across space and time appears an important stage for BDSA [5].

A Bloom Filter (BF) can be termed as a space-and-time efficient technique that was developed by Burton Bloom [6]. This attribute of BF reflects the prospect of employing this method for security analytics that supports monitoring the network stream [3, 4]. Even though eighteen BF types have been introduced as described in reference [7], only a few types are employed in the network security domain. In 2016, the BF was previously introduced for BDSA [8]. However, this study evaluated the performance of BF with a smaller dataset in order to only provide a proof of concept.

This paper, therefore, utilizes the BF with a large dataset for evaluating the performance and success rate of the proposed technique as well as proposing a novel approach for a false-positive rate reduction. The standard BF is selected, and the main reason behind choosing the standard BF is its involvement in the network security domain. Therefore, an experimental study is conducted using a high-performance machine with a dataset that is available in reference [9]. The results confirmed that the standard BF technique seems an applicable tool to address the data growth rate. However, the false positive of the proposed approach has a noticeable rate. Hence, a novel false positive reduction approach is proposed using a check bits' methodology. This approach is empirically evaluated, and the results are encouraging in terms of reducing the false positive rate (FPR).

The first contribution of this research is to evaluate the standard BF technique with a large dataset [9] and demonstrate its performance and success rate. Our second contribution is to address the issue of the false-positive rate and decrease it by introducing a novel false-positive reduction approach using check bits' methodology as explained in Section 7.

The rest of the paper is organized as follows: the literature review is presented in Section 2, whereas Section 3 details BF and its types. The big data definition is provided in Section 4.  Section 5 explains the experiments and the dataset. The results are illustrated in Section 6. The next section shows how the false-positive rate is reduced using the check-bits approach, whereas a discussion is provided in Section 8. Finally, the conclusion is given in Section 9 with future work.

## 2. Related Work

The only related work, as far as we know, is the study in reference [8] that applied the counting BF with a smaller dataset. Despite the fact that the study provided a proof of concept using the counting BF with a smaller dataset, our study extends the concept with a large dataset using the standard BF, as will be explained in Section 5. Thus, this section highlights BF and its application as well as big data security analytics.

Recent work suggests enhancing BF filters by using a prefilter based on machine learning for learning a function that can then model a data set the BF is meant to represent. Mitzenmacher [10] models such BF with the following outcomes: (1) What assurances can and cannot be attained with such a structure? (2) Assessing what scope the hashing function must acquire for amended performance? (3) A sandwiching approach for heightening learned BF (4) A design and analysis approach for a learned Bloomier filter, based on this learned BF.

Similarly, the authors in reference [11] suggest a novel machine-learning (neural network) based memory architecture, the neural BF (NBF), which can attain substantial compression gains over classical BF and prevailing memory-augmented neural networks and substitute data structures that have been created manually.

Christen et al. [12] argue that the BF's encoded values have vulnerabilities to cryptanalysis attacks. In the simulation, the novel efficient attack is carried out on BFs, which shows that these attacks are successful and thus repeatedly occurring sensitive attribute values can be re-identified. Therefore, the authors simulate and analyze the novel efficient attack on real databases that is founded on the construction principle of BFs of hashing elements into bits' positions. The proposed scheme is independent of the encoding function and can re-identify sensitive encoded values in a few seconds.

One-Hashing BF (OHBF) approach is presented by researchers in reference [12] because of its simplicity and as it requires one base hash function and some modulo operations. The overheads encountered due to using hash functions are mostly low despite keeping the same theoretical false-positive ratio as in the standard BF (SBF), hence increasing its performance.

To identify repeatedly co-occurring bits' position in the set of BF, a novel attack method-based technique is introduced [13], which is referred to as the frequent itemset mining technique. Although the encoded item set in the database is unique, still these proposed attack methods can re-identify sensitive encoded values. After analyzing and testing these proposed attack methods on various real-world problems, it effectively shows the re-identification of values from the encoded BF, even in such situations where the earlier attacks failed in the process.

An enhanced version of BF known as ultrafast BF (UFBF) is proposed in reference [14] by taking advantage of the single instruction multiple data (SIMD) processor approach. First, to speed up the membership checking process, three advancements have been made in UFBF. An improved novel hash computation algorithm has been developed that uses SIMD instruction to apply multiple hash functions in parallel resulting in reduced execution times. Second, BF's bit-test process is set to work in parallel, enabling it to carry out an increased number of bit-tests per unit time. Third, the efficacy of the membership check is improved by encoding a bit's information into a small block to be suitable for the cache. In the overall analysis, UFBF significantly outperforms state-of-art BF variants' membership checking speed.

On the other hand, big data indexing techniques have been enhanced in terms of accuracy performance. In particular, recent studies focus on optimizing search performance in big data with a better time-and-space tradeoff. Accordingly, a survey conducted in reference [15] reports the indexing for large datasets and has evaluated and classified indexing techniques into three classes: artificial intelligence (AI), non-AI (NAI), and collaborative AI (CAI) methods. NAI is the traditional approach, such as graph query processing, in which the indexing construction is straightforward and query responses. It performs practically in volume, velocity, variety, variability, value, and complexity. However, it could not detect the big unknown data. This unknown challenge is overcome by AI, such as a case-based data clustering technique that can be integrated with a fuzzy decision tree to develop a hybrid model that produces efficient results for test data.

Patgiri [16] developed a high-accuracy bloom Filter, HFil, a combination of several 3D BFs, to achieve high accuracy and reduced false positive rate. The best configuration achieved an accuracy of 99.99%. Kiss et al. [17] developed a novel data structure called EGH filter, supporting bloom filter operations and devoid of any false positives (mentioned as a false positive free zone).

Rottenstreich et al. [18] developed memory-efficient bloom filters also with a false positive free zone and discussed the role of Orthogonal Latin Square codes. Patgiri et al. [19] developed 3D BF specifically to manage passwords. Their approach got an accuracy of 99.99% and only 0.000001882 false positives. Najam et al. [20] used multiple BF (MBF) for DNA sequencing, whereby MBF stores the chromosome data. The false probability rate is kept at 0.01. The experiments showed that the FP plays an important role in compression, and a higher FP is able to get better compression.

**Figure 1:**
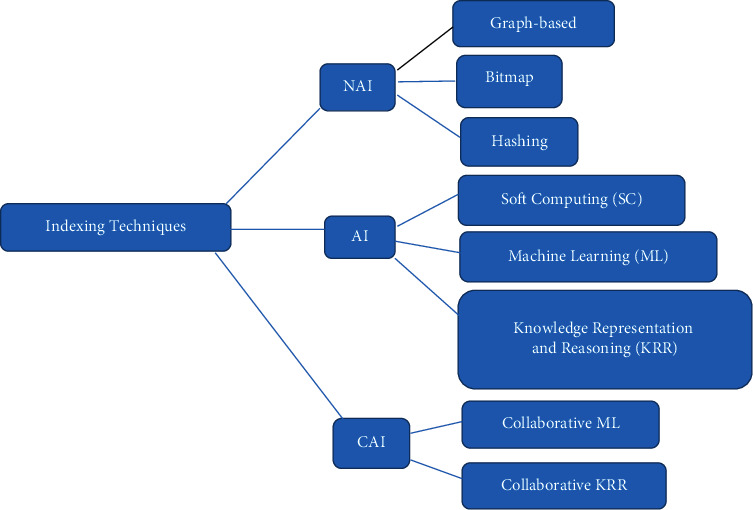
Summary of state-of-the-art big data indexing techniques.

Figure 1 summarizes the state-of-the-art big data indexing techniques based on previously mentioned categories.

Although there exists a considerable amount of related work considering a BF as an indexing technique, these formulations do not fit exactly with our approach.

## 3. Bloom Filter (BF) Technique

This section gives an overview of the BF and its types.

### 3.1. Bloom Filters: An Overview

Generally, a compact indiscriminate data structure to help membership queries is inferred as a BF [6, 21]. Even though the BF accumulates several elements in compact memory, a poor rate of false positives may arise in membership queries. On the contrary, the features of BF, i.e., space-saving and locating time, frequently harmonies the downside when the ratios of false positives are reduced.

Formerly, BFs have captivated vast notoriety in innumerable networking applications, namely, security and web caching [11]. The idea of SBF is explained as follows [22]: It allocates a bit vector *B* of *m* bits, each initialized with zeros and then chooses *k* independent hash functions, given as *h*_1_,…, *h*_*k*_, each having a range {0,…, 1 − *m*}. In order to add an element *s* into *B*, the bits at location *h*_1_(*s*),…, *h*_*k*_(*s*) in *B* are made equal to 1, i.e., make *B*[*h*_1_(*s*)=⋯=*B*[*h*_*k*_(*s*)]=1.

It is pertinent to mention that some bits could be set to 1 several times in which some added elements might share the same bits. Based on this, to query whether, for example, *s* is a member or not using the BF, the bits at location *h*_1_(*s*),…, *h*_*k*_(*s*) are verified. Accordingly, if all of these bits are one, then *s* is a member. Otherwise, *s* is not. However, there is a probability that all of these bits are one *butx* is not a part of set *B* (mentioned as “false positive”).

A false positive refers to returning “yes” while looking for an element that is not present in the filter. Nevertheless, getting a false-negative (returning “no” when querying an element that is present) is not possible. The probability that false positives can arise is given next:

(1) $$\begin{array}{c} f\approx {\left(1-{\left(1-\frac{1}{m}\right)}^{kn}\right)}^{k}\approx {\left(1-e^{\frac{-kn}{m}}\right)}^{k}. \end{array}$$

A tradeoff exists between *m* (storage size), *k* (computation time), and *f* (FPR). It could minimize *f* when *k*=ln 2 × m/n, where it becomes *f*_mtn_=(1/2)^*k*^ ≈ (0.6185)^m/n^. Therefore, as *m* increases in proportion to *n*, *f* will decrease [23].

### 3.2. Types of BF

Based on the applications, different flavors of BFs have been proposed. An upgradation to the standard BF is inferred as the Bloomier filter in reference [6]. In contemplation of subordinate values in the BF with a subcategory of the elements, the category can be prearranged with arbitrary functions. These functions are widespread while upholding their fiscal use of storage. In addition to this, dynamic updates are permissible; consequently, the functions persist unpretentiously. As we mentioned previously, eighteen BF types had been proposed in the literature [7]; nevertheless, few types have been used in the network security area. Thus, eight types that contributed to the network security field are discussed.

#### 3.2.1. Standard BF (SBF)

The SBF is explained in Section 3.1. Also, this type is utilized in this paper; more details are given in Section 5.

#### 3.2.2. Bloomier Filter (BLF)

BLF stores the membership function of the element set *S* : *f*=*S*⟶[0,1] as an enhancement to the SBF [15]. In particular, it encodes arbitrary functions to associate values with a subset of the elements.

#### 3.2.3. Compressed BF (ComBF)

Since the SBF has three main factors (i.e. *k*, *m*, and *n*), this type introduced another factor for the data size that is transmitted over the network called transmission size *z* [10]. The ComBF has a significant bandwidth saving.

#### 3.2.4. Counting BF (CouBF)

The CouBF type is developed by the authors of reference [24] as an extension of the SBF to count the occurrence of e-mail messages. This type is upgraded by the authors of reference [23] in order to know the number of times an e-mail message has been added to the BF.

#### 3.2.5. Dynamic BF (DBF)

The DBF type addresses the issue of knowing the size of a static set by creating a dynamic set *A* with a dynamic *n* × *m* bit matrix. That is, an SBF is initially activated (i.e., *n*=1). In case the FPR is increasing, a new SBF is activated, and the older one becomes the current one [25].

#### 3.2.6. Generalized BF (GBF)

The GBF type addresses the issue of lacking the upper bound in the FPR of the SBF by putting an upper bound on the false positive probability [26]. This type is appropriate for security purposes.

#### 3.2.7. Hierarchical BF (HBF)

This type has two levels of probabilistic arrays [27]: a probabilistic array when the accuracy is low, and another one when the accuracy is high. Using this type, the performance and scalability of the file systems are efficiently enhanced.

#### 3.2.8. Space-Code BF (SCBF)

The SCBF type measures per-flow traffic approximately [28] to answer queries of the form whether an element *x* is in a multiset.

Table 1 summarizes these types and their contributions in the network security domain [8].

## 4. Big Data

The term big data is related to a collection of data from different sources in which numerous datasets can be produced. Hence, a real challenge is observed due to the size and time of analytical effort in analyzing the data set. This section, therefore, describes the characteristics and indexing approaches for big data.

### 4.1. Big Data Characteristics

Four common features of big data, namely, volume, variety, velocity, and veracity, are defined as follows:

- Volume: It refers to huge datasets, i.e., terabytes to exabytes of stored information, in areas such as social networks
- Variety: Data are shaped from numerous sources, for example, unstructured, structured, and semi-structured data
- Velocity: This reflects the speed of receiving and sending data
- Veracity: This refers to sufficiently accurate data, not spoofed data, or not corrupted data.

### 4.2. Big Data Indexing Techniques

The characteristics mentioned earlier may negatively impact existing indexing solutions in terms of their solutions becoming ineffective. For this [1], classified indexing techniques into three categories, as shown in Figure 1, and concluded that the CAI technique is the most effective for big data.

## 5. Experiments

This section describes how we built the BF and performed its evaluation by conducting an experimental study.

### 5.1. Building the SBF

We first define the main class for the SBF that encloses methods that perform BF-related operations, such as hashing, adding, checking, searching, and other necessary helper functions. In the beginning, a constructor is built which initializes the BF object to receive the following:

- The total number of items to store in the SBF (*n*) which we use to compute the SBF size *m*.
- The false-positive probability (*f*_p_prob_) value, which is a preset probability of getting a false positive (when checking whether an item has been inserted into the filter or not). Lowering the *f*_p_prob_ value increases the confidence level in the SBF giving us fewer false positives if queried for items non-existent in the SBF.
- An item type, dependent on which particular items to store in the SBF. It could be user information, computer information, timestamp information, or all.
- A hash count value asserts the number of hashing operations to perform when adding and searching for items in the SBF.

Inside this constructor, we have instantiated the SBF as a bit-array, an array-like data structure that compactly stores bits (0's and 1's) or Boolean values. All bits are set to 0 at the point of instantiation.

#### 5.1.1. SBF Size

To compute the size of the SBF based on and value, a method is defined as shown in equation (2).

(2) $$\begin{array}{c} m=-\frac{n\;\log\left(f_{p\_\text{prob}}\right)}{\log\;{\left(2\right)}^{2}}\text{.} \end{array}$$

#### 5.1.2. Number of Hash Functions

The number of hash functions *k*, depend on the computed BF size *m*, and total number of items to be added *n* as depicted in equation (3):

(3) $$\begin{array}{c} k=\frac{m}{n}\log\left(2\right)\text{.} \end{array}$$

#### 5.1.3. Hashing

To generate *k* hash values, a method is called for every single BF addition. Given an input value of arbitrary length, such a method outputs *k* different values, which will always be unique to the input value and smaller or equal to the SBF size.

#### 5.1.4. Adding

Using the hashing approach described earlier, we obtain *k* unique values for an item to be added or stored. Thereafter, we access bits in locations/indices (corresponding to *k* unique values) within the bit-array and change them from 0 to 1.

#### 5.1.5. Searching

Similar to the aforementioned adding process, the hashing is used to obtain the *k* unique values for an item to be looked up, thereafter, access and check, if all bits in the locations/indices (corresponding to the unique values) are 1 s. If we find any bit as 0, this function returns *false* (in other words, the item is absent); otherwise, all checked bits have to be 1 for the function to return *true* (implying the item is present or exists).

### 5.2. Used Dataset

We used an anonymous dataset in this research named “User-Computer Authentication Associations in Time” [9]. The data contain 708,304,516 authentication events from an enterprise computer network. The events are recorded for nine continuous months, whereby an event corresponds to successful user authentication to a particular computer at a certain time. The data are accessible both as a single file and in the form of nine files, one for each month. The number of distinct users is 11,362, whereas 22,284 computers are available to users. There are three attributes, namely, timestamp, user information, and computer detail. The timestamps do not represent the actual time, but start at 1 and represent the offset. This arrangement makes the data fully anonymous.

The researchers in reference [9] used this dataset to generate authentication graphs and studied the size of the largest connected components. They further discussed that their models could be employed to limit or even remove highly connected computers and users. The justification provided by them is that the computers which are used by a relatively large number of people need additional security.

### 5.3. Evaluating the SBF

Having implemented the SBF in Section 5.1, the next step is to evaluate the SBF and examine its ability to perform SBF-related operations. Each particular item (user, computer, or timestamp) stored in the filter and representing the element to find within the dataset (described next) needs to be specified.

#### 5.3.1. Default Process

The default implementation of evaluating the BF is determining the number of items that are going to be added to/stored in the filter, a false positive probability, and a particular item which can either be a user, computer, timestamp, or all of them (dataset properties). The prompt responses are used to instantiate BF to perform its operations, such as creating a list of items to add and search for in the SBF, adding items to the SBF, and searching for items in the SBF.

#### 5.3.2. Evaluating SBF Properties

The memory, adding time, searching time, and false-positive rate versus false probability are explained in the following:

*(1) Memory and Number of Items*. This is an evaluation of how the memory (SBF size) *m* varies with an increasing number of items (*n*) to store in the SBF. This involves iterating through a range of different *n* values, i.e., different numbers of items to store, and for each iteration, the SBF is instantiated with a default false positive probability value and a default item (user, computer, timestamp). Given the number of items *n* and false-positive probability, SBF size is accordingly computed as shown in Section 5.1.1. Each computed BF size is stored in a dictionary along with the corresponding *n* value, which is later converted to a CSV file. We additionally compute the rate at which the SBF size changes from one *n* value to another, given the entire list of computed BF sizes.

*(2) Adding (Saving) Time*. Adding time is an evaluation of the time it takes to add or store items. This time varies with an increasing number of items (*n*) to store in the SBF. Given the items, we iterate through a range of different values that are multiples of 40 (a hyperparameter that is randomly chosen and subject to change). For each, a list of items to store is created, and *n* different items are added to the SBF as described in Section 5.1.4. A dictionary is filled with various *n* values and the time taken to add all items (which is computed as the time at the end of the item addition minus the time stored before the addition operation is called). This time is computed for each iteration. The dictionary is later converted to a CSV file with the number of added items and the corresponding filling time.

*(3) Searching Time*. The searching time is the time it takes to look up items in the SBF. This time varies with an increasing number of items to search in the SBF. This begins by filling the SBF with items. It then iterates through a range of values that are multiples of 40 (can be any other alternative). For each iteration, a list of items is created to search as a combined list of items. A dictionary is then filled with each list and the time taken to search all items, which is computed as the time at the end of the search of the item minus the time stored before the search operation is called. This dictionary is later converted to a CSV file with the number of searched items and the corresponding searching time.

*(4) False Positive Rate and False Probability*. This evaluates how varying the false positive probability affects the FPR while searching the SBF. The process of searching time is repeated with a range of false-positive probability values in the range of 0 to 1. For each of these probabilities, we store the average FP count while searching items. Each time the check function incorrectly indicates the existence of an item, the FP count is incremented by 1. As such, *X* different FP counts are obtained for *X* corresponding groups/lists of items searched, and therefore the average FP count is computed. The FP rate is obtained as the percentage of the total-searched items that the average FP count is. A dictionary is filled with each tested FP probability value and the computed average FP counts. This dictionary is later converted to a CSV file with the number of searched items and the corresponding searching time.

*(5) Hash Functions with Filling and Searching Time*. This experiment evaluates how increasing the number of hash functions varies with the time it takes to add in items and look up items in the SBF. It varies with an increasing number of items to search in the SBF. Using a fixed number of items to add to the SBF and a fixed item type such as a user, the process is to iterate through a range of hash count values, in which case the SBF is instantiated with the two fixed values and the hash count value at hand. During each iteration, two lists are created, the first including items to add to the SBF, whereas the second includes out-of-sample list items. The SBF is filled with all items in the first list and subsequently searched for items in the second list and 25% of the items in the first. The time each operation takes is recorded for each hash count value, and all these are stored in a dictionary, which is later converted into a CSV file.

## 6. Results

The results of testing the false positive and the memory and time consumption are presented in this section.

### 6.1. Testing the False Positive

**Figure 2:**
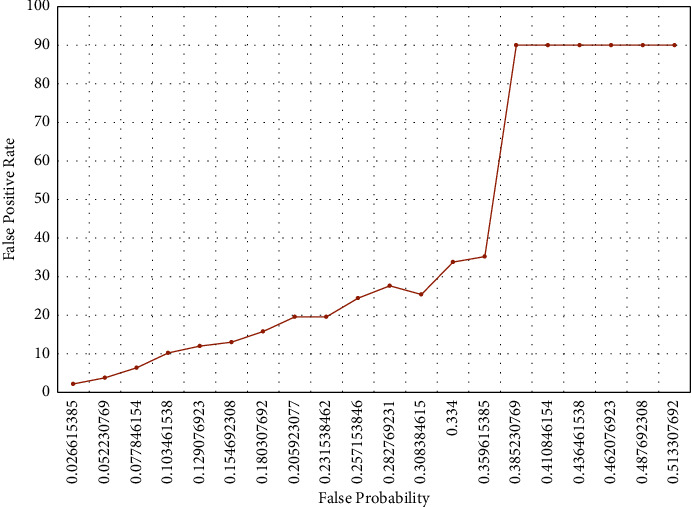
False positive rate vs. false probability for BF.

Figure 2 shows the graph for false-positive rate (FPR) vs. false probability for the implemented SBF. A steady increase in the FPR is observed as the false probability increases from 0.027 to 0.206. Similarly, a rapid increase (155.68%) in FPR could be seen as the false probability increases beyond 0.360. However, no change in FPR is observed as the false probability becomes greater than or equal to 0.385. This graph shows that the lower the false probability, the lower would be the FPR. Furthermore, a lower value of FPR is required in all types of experiments related to the BF.

### 6.2. Testing the Consumption of Memory

**Figure 3:**
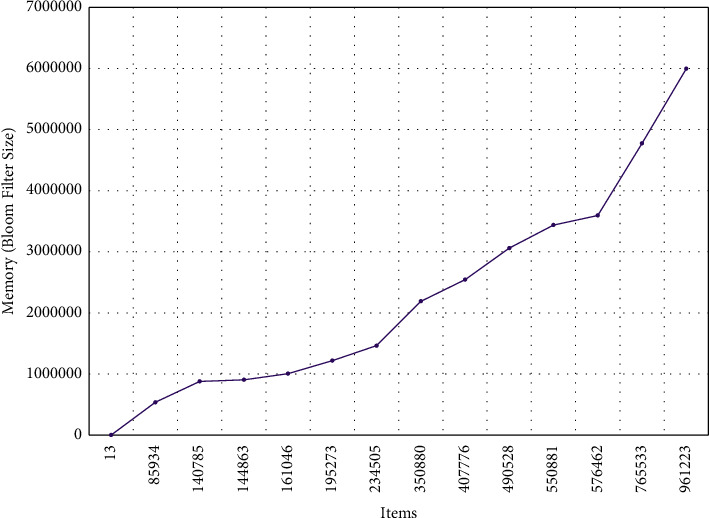
Change in memory requirements as the number of items increases.

Figure 3 provides a complete analysis graph of the memory requirements as the number of items is increased from 13 to 961,223. Consequently, BF size increases from 81 to 5,993,440. The memory represents the size of the BF, and an overall increase in memory requirements is observed in the graph. Nevertheless, the rate of change varies as the number of items increases. A considerably greater BF size (4,773,269 for 765,533 items) is required as the number of items increases beyond 576,462. A 32.8% increase in memory is required as the number of items is increased from 576,462 to 765,553. On the other hand, the required memory does not increase at a greater rate as the items are increased from 140,785 to 234,505.

### 6.3. Testing the Consumption of Time

**Figure 4:**
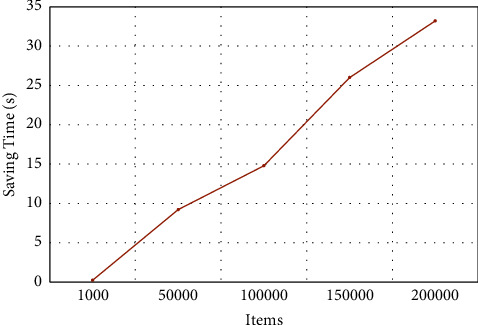
The impact of changing the number of items on saving/adding time.

Figure 4 illustrates the effect of increasing the number of items on time required by the BF. One can observe that as the number of items increases from 1000 to 200,000, the time required by the BF increases. Nevertheless, the increase is not linear, and the rate of change in time is not uniform, e.g., the rate of increase is lower when the number of items is varied from 50,000 to 100,000, as compared to the change in items from 1,000 to 50,000 or from 100,000 to 150,000. In the latter case, as the number of items increases from 100,000 to 150,000, an increase of 75% in time is observed. On the other hand, only a 27.77% increase in time is seen as the number of items increased from 150,000 to 200,000. Thus, 33.21 seconds are required while dealing with 200,000 items instead of just 0.238 seconds for 1000 items.

**Figure 5:**
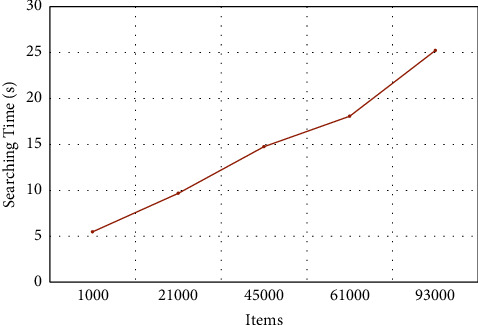
Impact of changing the number of items on the searching time.

Figure 5 shows the impact of modifying the number of items on time required for searching in a BF. In general, the time required for searching for an item in a BF is directly proportional to the number of items. However, a different rate of change is observed as the number of items increases from 1000 to 45,000 as compared to the increase from 61,000 to 93,000. An increase of 39.57% in time is seen as the number of items increased from 61,000 to 93,000. While 5.482 seconds are required for searching 1,000 items, 25.235 seconds (more than four times) were needed to search 93,000 items in the BF. Comparing Figure 5 with Figure 4, one can observe that the time for searching is more than the time required for adding items in the BF.

## 7. Reducing the Number of False Positives: Check Bits' Approach

Due to the observed increase of the FP rate in our previous experiments, we have proposed an approach in order to reduce it. In particular, the proposed algorithm for reducing false positives in the BF is based on two check bits. The input element to the BF, which can be in numerical or textual form, is converted to the binary form. Then, all the bits are summed up to come up with a number in decimal form. Later, the number is again converted to the binary form. The next step is to determine the two check bits. For this purpose, we find the middle bit of the binary equivalent of decimal representation and take one bit located five places to the left and another one from five places right of the middle bit. With initial experimental evaluation, we found that the five-position displacement for taking the two bits produced improved results compared to other possibilities.

For the proof of concept, we conducted a preliminary set of experiments. The obtained results are presented in Table 2. We tested our approach based on check bits with various BF sizes, varying from 25 to 100. The number of hash functions and the checked items is kept fixed as 2 and 350, respectively. When the size of the BF is 25, the number of FP without any additional check bits is 256. However, by adding just two check bits, the FP was reduced to just 71. This represents a 72.27% reduction in the FP rate. Similarly, as the BF size is increased to 50, the FP rate without using any check bits is 272. In this case, the number of FP while employing check bits is just 74. This scenario shows an 81.62% reduction in the FP rate. Lastly, as the size of the BF increases further to 100, the FP rate was observed to be 288 while ignoring any check bits. Nevertheless, the FP rate with check bits was reduced to 84. The reduction in the FP rate amounts to 70.83%.

**Figure 6:**
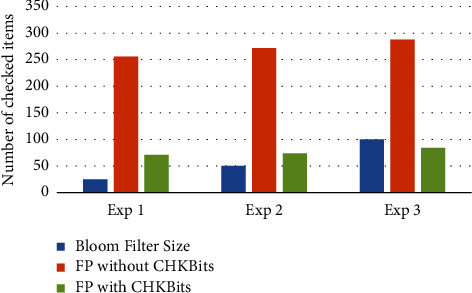
Results of the three experiments for the proof of concepts of reducing the false positives. The orange bars represent the false positives without the check bits. The green bars show the false positives using the check bits and, thus, a reduction of the false positives. (Exp: experiment, CHKBits: check bits).

Comparing three scenarios, the second scenario presents the maximum reduction in the FP rate. The results of the three experiments for the proof of concept of reducing the false positives are also represented in Figure 6. In Figure 6, the orange bars represent the false positives without check bits. The green bars show the false positives using the check bits, and thus, a reduction of the false positives. Khan et al. [29] studied the impact of changing the number and position of check bits on the number of false positives. Furthermore, they also found that an increase in the bloom filter size, in general, reduces the false positives.

## 8. Discussion

The obtained results show an impressive performance level and success rate for applying the SBF in overcoming the efficiency lacking in the space-and-time of indexing and analyzing big data.

Specifically, it is observed in Figure 3 that SBF size increases as a reaction to the increased number of items. This reaction, however, varies as the number of items is increased. That is, the SBF size of 4,773,269 is required for 765,533 items, while 961,223 items need 5,993,440 SBF size. Remarkably, when the items are increased from 140,785 to 234,505, the SBF size is stable (i.e., not increased). Moreover, although there was an impact on the time consumption due to the increase in the number of items, this increase is not linear, and the rate of change in time is not uniform, as shown in Figure 4.

Similarly, Figure 5 shows the increase in searching time as the number of items increased. The growth in time is steady but not linear. While comparing Figure 4 with Figure 5, one can quickly note that more time is required, in general, for searching as compared to the time needed for adding items in the BF. Whereas only 14.98 seconds are required to add 100,000 items in the BF, 25.2 seconds are necessary to search for an item in a BF having 93,000 items.

Figure 2 shows a steady increase in the FPR, as the false probability increases from 0.027 to 0.206. This increase, however, is not observed while the false probability becomes greater than or equal to 0.385. Furthermore, since our paper focuses on big data security analytics, the FPR is different. That is, for example, in the intrusion detection system, the FPR refers to a normal activity that mistakenly is identified as attack behavior, while the false-negative rate (FNR) refers to an attack activity that is identified as a normal activity. Accordingly, the possible correlation between the FPR in the SBF and the FPR in the cyber security domain is that an attack activity is found, while it truly does not exist in the system. Although the FPR does not influence the security level generally, the results would be more accurate when reducing the FPR. For this, we proposed a novel FPR reduction approach in which the check bits are exploited. The results of evaluating this approach appear very interesting. Nonetheless, a comprehensive experiment with a larger dataset will be conducted as one of our future works.

It is noteworthy that the Cuckoo filter, like the BF, is a space-efficient probabilistic data structure. However, the main difference between the Cuckoo filter and the BF is that the Cuckoo filter can delete existing items, while the BF cannot. A work by the authors of reference [30] demonstrated a comparison between the Cuckoo filter and the BF in terms of FPRs. The results of this study showed that the Cuckoo filter provides better practical performance, under certain circumstances, but ultimately, it can serve as an alternative in scenarios where a BF would normally be used.

To meet the rate of data growth, managing big data appears a challenge. Nevertheless, the SBF type demonstrates the ability to cater to this growth effectively because to its dynamic feature for size.

## 9. Conclusion and Future Work

This paper evaluates the performance of a memory-and-time efficient probabilistic approach empirically for big data security analytics. That is, the standard bloom filter technique (SBF) is selected and implemented due to its contributions to the network security field using a large dataset. To evaluate the proposed approach, an experimental study is conducted. The results showed an interesting performance in terms of overcoming the effectiveness in the space and time of indexing and analyzing big data. Although the false positive with SBF has a conspicuous rate, this would not impact the system's security level. To address the increase of the false-positive rate, a novel false-positive rate reduction method is introduced and implemented. The initial results of evaluating this method were promising.

A possible future work is to evaluate the proposed false positive rate reduction method employing a larger dataset. Furthermore, a comparison could also be made between the Cuckoo filter and the BF in terms of memory and time consumption.

## Figures and Tables

**Table 1:** Summary of BFs types and their contribution to network security.

By type	Authentication	Firewall	Anomaly detection and privacy-preserving	Traceback	Node replication detection	String match	Email protection	SYN flooding addressing
SBF	^*∗*^	^*∗*^	^*∗*^	^*∗*^	^*∗*^	^*∗*^	^*∗*^	^*∗*^
BLF						*∗*		
ComBF	^*∗*^			^*∗*^				
CouBF		^*∗*^				*∗*	*∗*	*∗*
DBF					^*∗*^			
GBF				^*∗*^				
HBF				*∗*				
SCBF				*∗*				

**Table 2:** The preliminary results of the three experiments executed for the proof of concepts of reducing the false positives. For all three experiments, the false positives are considerably reduced.

BF size	Hash functions	FP without CHKBits	FP with CHKBits	Number of inserted items	Number of checked items
25	2	256	71	25	350
50	2	272	74	50	350
100	2	288	84	100	350

## Data Availability

The dataset could be made available on request to the corresponding author.
